# Ocular therapeutics and the profession of optometry in South Africa

**DOI:** 10.4102/phcfm.v16i1.4140

**Published:** 2024-01-16

**Authors:** Rekha Hansraj, Nokulunga Dlamini, Shoaib Khan, Phumelele C. Mtolo, Nqobile G. Ntuli, Cherise Prithipal, Husnaa Salajee, Zamadonda N.Q. Xulu-Kasaba

**Affiliations:** 1 Department of Optometry, College of Health Sciences, University of KwaZulu-Natal, Durban, South Africa

**Keywords:** ocular therapeutics, certification, optometrists, primary eye care, prescribing, optometrists, South Africa, clinical training

## Abstract

**Background:**

The role of an optometrist as defined by the World Council of Optometry includes the management of ocular diseases. In 2015, the scope of optometry in South Africa was expanded to include ocular therapeutic drugs. To date approximately 27 optometrists have obtained full certification to exercise ocular therapeutic privileges.

**Aim:**

This study aimed to determine the interest, readiness, as well as challenges, of optometrists for the inclusion of ocular therapeutics into daily practice.

**Setting:**

The study was set in South Africa.

**Methods:**

A descriptive, cross-sectional study design was employed. Convenience sampling was used to recruit 420 participants from a study population of optometrists registered with the Health Professions Council of South Africa, with data collected using an online questionnaire hosted on social media platforms and distributed by professional organisations. Data were analysed using the Statistical Package for Social Science version 27.

**Results:**

The majority of respondents (73.3%) reported keenness for ocular therapeutics certification. While 75.7% of respondents had obtained diagnostics certification, only 9.5% were registered with the Health Professions Council of South Africa (HPCSA) with ocular therapeutics certification. Most (92.1%) respondents reported the required minimum of 600 h of clinical training as a major challenge to obtaining ocular therapeutics certification. Almost all (96.9%) of the respondents agreed that ocular therapeutics certification will improve provision of optimal eyecare.

**Conclusion:**

South African optometrists support and are personally interested in ocular therapeutics certification. However, while there is a preponderance of diagnostically qualified optometrists, very few are certified for ocular therapeutics with completion of the required clinical training for certification perceived as the greatest challenge.

**Contribution:**

This findings in this study highlight that, current requirements to support ocular therapeutics certification of South African optometrists should be reviewed to ensure an enabling environment for the completion of the clinical training.

## Introduction

An optometrist, as defined by the World Council of Optometry (WCO), is a primary eye care practitioner involved with the diagnosis and management of refractive error and ocular diseases, as well as the rehabilitation of the visual system.^1^ This definition was adopted by the WCO in 1993 taking into consideration the legislative differences in the scope of optometric practice around the world. In most countries, optometrists are legislated for the practice of optical technology, visual function and ocular diagnostic services, which are levels 1, 2 and 3, respectively, of the Global Competency-Based Model of Scope of Practice in Optometry.^1^ Ocular therapeutic services (level 4) is also considered as a minimum competency in the global model, yet only optometrists primarily in the developed countries including the United Kingdom (UK), United States of America (USA), Canada, Australia, New Zealand and Norway have long been legislated for its inclusion in practice.^1,2^ In Africa, previously it was only in Nigeria and Ghana that the full scope of optometry was practised.^3,4^ In South Africa, the scope of optometry has now been expanded to include ‘the use of scheduled substances as approved by the board and Medicines Control Council’ (No. 29174, Government Gazette, 8 September 2006) with legislation for level 4 only received in 2015 and the first schedule of approved pharmaceutical agents for use by optometrists with ocular therapeutics released in 2016.^5,6^

The importance of ocular therapeutics inclusion in the delivery of eye care cannot be overstated considering the global burden of eye disease. In sub-Saharan Africa up to 25% of the population is affected by eye disease with eye care provided primarily by ophthalmologists, optometrists and ophthalmic nurses but in the ratio of approximately 1:4:3, respectively.^7^ In addition to the vast difference in numbers particularly of ophthalmologists and optometrists, the distribution of eye health providers is skewed, with most of them located in urban areas, while many people who are visually impaired often reside and seek eye care in the rural areas.^8,9^ About 33.7% of optometrists in sub-Saharan Africa are employed in small towns and rural areas.^10^ Similarly, in South Africa, there is a predominance of registered optometrists (4204) in relation to ophthalmologists (582) (Daffue Y 2023, personal communication to Hansraj R, April 19, 2023). Even though the majority (93.3%) of optometrists serve in the private sector, an estimated 6.7% are employed in the public sector where an ophthalmologist is not always available.^11^ Thus, the therapeutic endorsed optometrist is expected to be most beneficial in the delivery of eye care in the country. In essence, prompt management of certain eye diseases can be ensured especially in rural areas negating the need for referrals, which could enable ophthalmologists to deal with other eye conditions particularly those requiring surgical intervention, thereby reducing the increasing burden of ocular diseases, for example, untreated cataracts, that the country faces.^12,13^

To meet the needs for the expanded scope of optometry in South Africa, the Post Graduate Certificate in Ocular Therapeutics (PGCOT) was introduced in 2015 through the collaboration of the State University of New York (SUNY) and the University of KwaZulu-Natal (UKZN).

To obtain the certification to use ocular therapeutic drugs, optometrists who are certified to use diagnostic procedures are required to complete a theoretical component consisting of three modules, two focussed on ocular disease management, ocular pharmacology and systemic conditions and one on ethics and public health. On completion of the theoretical component, optometrists can then commence with completion of a minimum of 600 clinic hours, which entails the prescribing of ocular therapeutic drugs under the supervision of an ophthalmologist or medical officer at a public sector ophthalmology department. Evidence of completion of the required minimum clinic hours is provided in the form of logsheets detailing the ocular condition managed with therapeutics signed off by the supervising practitioner and bearing the hospital stamp. This portfolio of evidence, which should also include case reports on at least 10 interesting cases, is submitted to the accrediting institution for approval. On successful completion of the clinical component a certificate is issued to the optometrist by the institution, which they use to get official recognition and permission from the Health Professions Council of South Africa (HPCSA) for the use of ocular therapeutics in optometry in South Africa.

Approximately, 135 optometrists were enrolled into the initial programme in 2015.^5^ Another similar course began in 2021 at the University of Johannesburg (UJ) with 102 optometrists enrolled (Hasrod N 2022, personal communication to Hansraj R, January 17, 2022). While about 218 optometrists have succeeded in completing the theoretical modules since 2015 (Moodley VR 2021, personal communication to Prithipal C, March 24, 2021), only 27 optometrists were registered with the HPCSA with ocular therapeutics by January 2022 (Daffue Y 2022, personal communication to Hansraj R, January 12, 2022). Thus, while in South Africa it has taken many years to reach the point of being legislated to practise the full scope of the optometric profession, there remain challenges preventing this expanded scope from being realised in practice. Anecdotal evidence suggests that while optometrists are keen on pursuing qualifications in ocular therapeutics, major challenges such as availability of training sites and ophthalmologists who are willing to serve as supervisors have been realised. This type of epidemiological information on the adoption of independent prescribing was also observed as lacking in the UK in a systematic review.^14^ This study therefore investigated the interest, readiness and challenges of South African optometrists in including ocular therapeutics in their daily optometric practice.

## Research methods and design

### Study design

A descriptive, cross-sectional and quantitative research design, utilising an online survey, was employed for this study.

### Study setting, study population and sampling strategy

The study population included optometrists within South Africa, registered with the HPCSA (*N* = 4040) (Daffue, Y 2022, personal communication to Hansraj, R, 03 November 2021). The minimum sample size, based on a total study population of 4040 optometrists, calculated using the Cochran formula was 350, which was increased by 10% to yield a minimum sample size of 385. Convenience sampling was used to recruit respondents for this study as the survey link was sent out through optometry social media groups (WhatsApp and Telegram) and non-governmental organisation (NGO) contact lists.

### Data collection

A structured, self-administered online survey form was used as the data collection instrument. This survey was designed by the researchers specifically to address the objectives of this study utilising Google forms for ease of online administration and distribution. The survey form comprised mostly closed-ended questions grouped according to the main areas explored in the study, which included the level of interest in ocular therapeutics, readiness of optometrists to include ocular therapeutics in their daily practice and the perception of optometrists of the challenges to obtaining ocular therapeutics certification in South Africa and its possible impact on the delivery of eye care. The survey piloted for face validity with the participants (*n* = 20) excluded from the main study.

### Data analysis

Data were captured and analysed using the Statistical Package for the Social Science (SPSS) version 27. As the data were captured manually by one of the researchers but verified by another to ensure accuracy, descriptive and inferential statistics were calculated, including percentages and 95% confidence intervals. Fisher’s exact tests, chi-squared test and rank-sum tests were used to test for associations between demographic variables and subject responses.

### Ethical considerations

Ethical clearance was obtained from the Humanities and Social Sciences Research and Ethics Committee of the University of KwaZulu-Natal (HSSREC/00002834/2021) prior to data collection and the tenets of the Declaration of Helsinki were adhered to where necessary. Informed consent was obtained in writing from all respondents. The identity of all respondents remained confidential and the research data were only accessible to the researchers through a password-protected computer.

## Results

### Demographics of respondents

**FIGURE 1:**
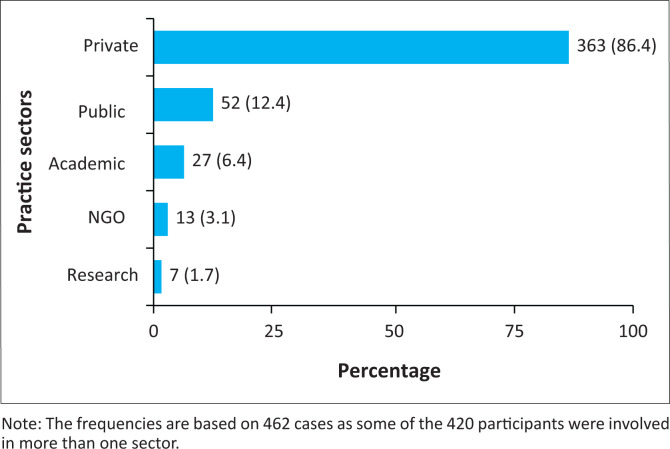
Practice sectors of respondents.

A total of 420 optometrists (*n* = 420) responded to the survey, reflecting a 10.4% response rate out of the total number of registered optometrists (*n* = 4040) at the time of the study. Approximately two-thirds of the respondents (66.7%) were female. The ages of the respondents ranged from 22 to 76 years, with a median of 36 years (interquartile range [IQR]: 29 to 46). Just under half of the respondents (44.8%; *n* = 188) had graduated with their Bachelor of Optometry (BOptom) degree at the UKZN, followed by UJ (22.4%), the University of the Free State (UFS) (11%), University of Limpopo (10.7%) and the previous Witwatersrand Technikon (6.9%). The majority (77.1%) of respondents had only a BOptom qualification, 3.6% had a BOptom and also a Bachelor of Science qualifications, and 5.2% had a Diploma in Optometry. In addition, 6.7% had a Master of Optometry (MOptom), 2.4% had a Master of Business Administration and 1.0% a Public Health masters. Ten respondents (2.4%) had obtained their Doctor of Philosophy qualification. Almost all the respondents (97.9%) are currently practising optometry in South Africa with the majority (86.4%) in the private sector (Figure 1).

Some respondents (10.7%, *n* = 45) had previously practised outside of South Africa including in the United Kingdom, USA and Namibia for durations ranging from 2 months to 14 years (majority for 2 years [2.4%]).

**FIGURE 2:**
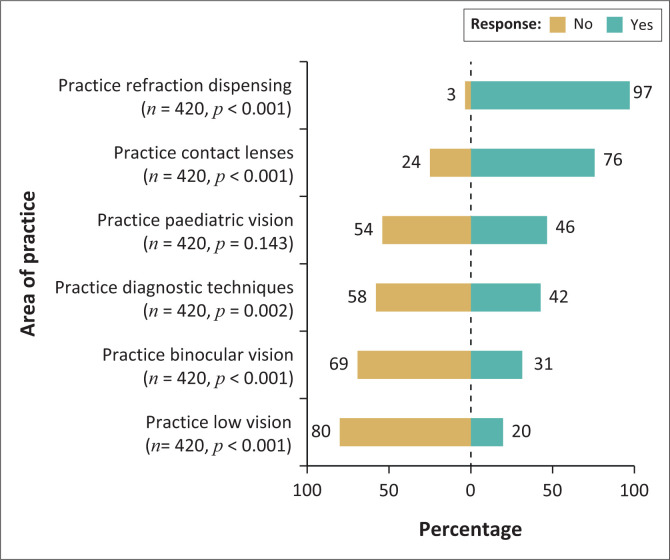
Areas of optometry practised routinely by the respondents.

The skills and competencies of optometry routinely practised by the respondents are illustrated in Figure 2. Significantly larger numbers of optometrists practised refraction, dispensing (97%) and contact lenses (76%), while specialist skills of low vision (LV) (20%), binocular vision (BV) (31%) and diagnostic techniques (42%) were practised to a significantly lesser extent (Figure 2). Even paediatric vision (PV) was practised routinely by less than half of the respondents (46%).

Even though 75.7% of the respondents (*n* = 318) were certified to have diagnostics skills and competencies, only 42% reported applying those routinely in their practice. A significant association was observed between diagnostics certification and age (*p* < 0.001; rank-sum test) with the younger optometrist more likely to be certified.

### Interest in ocular therapeutics

The overwhelming majority (97.4%) of respondents indicated that optometrists should be trained in ocular therapeutics and permitted with certification to prescribe pharmaceutical drugs with 90.7% reporting being confident to include ocular therapeutics skills in their daily routine. A slightly lesser number (*n* = 312, 74.3%) although were personally keen on obtaining their ocular therapeutics certification with 32 (7.6%) respondents indicating having already completed and 43 (10.2%) still in the process of completing, their ocular therapeutics certification. Age (*p* < 0.001; Kruskal–Wallis test), graduating institute for bachelor’s degree (*p* = 0.003; Fisher’s exact test), and highest qualification (*p* = 0.002; Fisher’s exact test) was found to be associated with keenness on ocular therapeutics certification.

**FIGURE 3:**
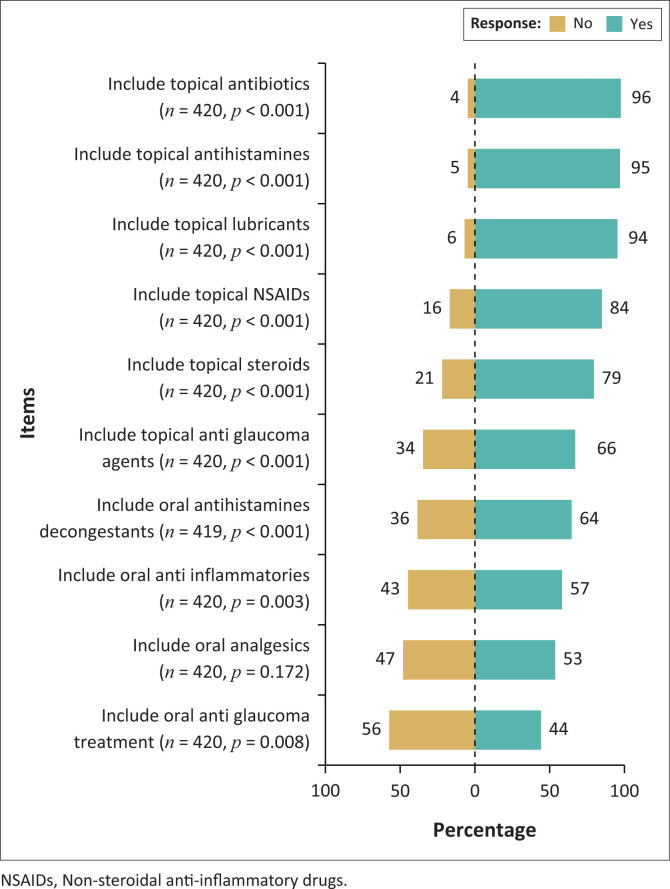
Respondents’ perceptions on agents that the ocular therapeutics certified optometrists should be allowed to prescribe.

Figure 3 illustrates the perceptions of respondents on ophthalmic agents, based on a list provided in the survey, that the ocular therapeutics-certified optometrist should be permitted to prescribe. With the exception of oral anti-glaucoma treatment, the majority of respondents supported the use of all therapeutic agents listed in Figure 3. There was more support for the use of topical rather than oral medication.

In addition to the use of therapeutic agents, a significant majority of respondents supported an expanded scope to also include foreign body removal (*n* = 399, 95%), scleral indentation (*n* = 344, 82%), punctal plug insertion (*n* = 344, 82%) and lacrimal dilation and irrigation (*n* = 294, 70%).

With regard to remuneration, 92.1% (*n* = 387) believed that ocular therapeutics-certified optometrists should be fairly remunerated for the additional qualification by means of increased consultation rates (*n* = 207, 49.3%) where applicable and/or procedure-specific claims (*n* = 355, 84.5%).

### Readiness of Optometrists for ocular therapeutics

**FIGURE 4:**
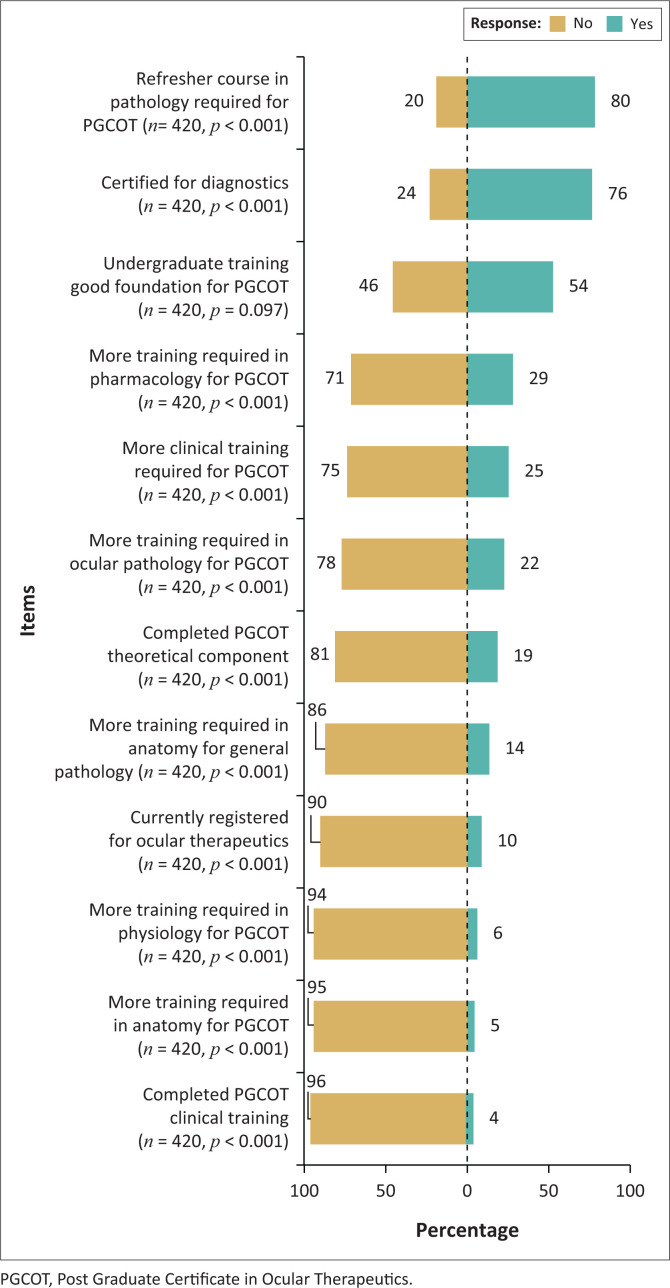
Readiness of optometrists in South Africa for ocular therapeutics.

Figure 4 details various aspects that impact the readiness of the South African optometrist for ocular therapeutics including ocular diagnostics and therapeutics in undergraduate training and postgraduate training and certification in ocular diagnostics and therapeutics. The items are arranged in the order of their frequencies of yes/no responses. The majority (*n* = 319, 76%) of respondents indicated that they have diagnostics certification with 69.5% (*n* = 292) of them having obtained it during their undergraduate training. Only 19.3% (*n* = 81) of respondents had completed the theoretical component of the PGCOT, with the majority of them (*n* = 251, 59.8%) having completed the theoretical component during the 2015–2016 offering of the programme. Moreover, of those who had completed the theoretical component, only 18.5% (*n* = 15) had completed the clinical training towards ocular therapeutics certification, where the majority (*n* = 308, 73.3%) had done so in the period 2018–2020. An additional 40 (9.5%) respondents were currently registered for ocular therapeutics. Out of all the respondents, only 9.5% (*n* = 40) were currently registered with the HPCSA as optometrists with ocular therapeutics privileges.

The majority of respondents (*n* = 336, 80%) believed that a refresher course in pathology was required before attempting the PGCOT despite only 22% being of the opinion that more training was required in ocular pathology. Over half of respondents (*n* = 227, 54%) felt that their undergraduate training provided a good foundation for the PGCOT. Furthermore, the majority of respondents did not feel the need for further training in pharmacology (*n* = 298, 71%), clinical training (*n* = 315, 75%), anatomy for general pathology (*n* =361, 86%), physiology (*n* = 394, 94%) and anatomy (*n* = 399, 95%) (Figure 4).

### Challenges to obtaining ocular therapeutics certification

**FIGURE 5:**
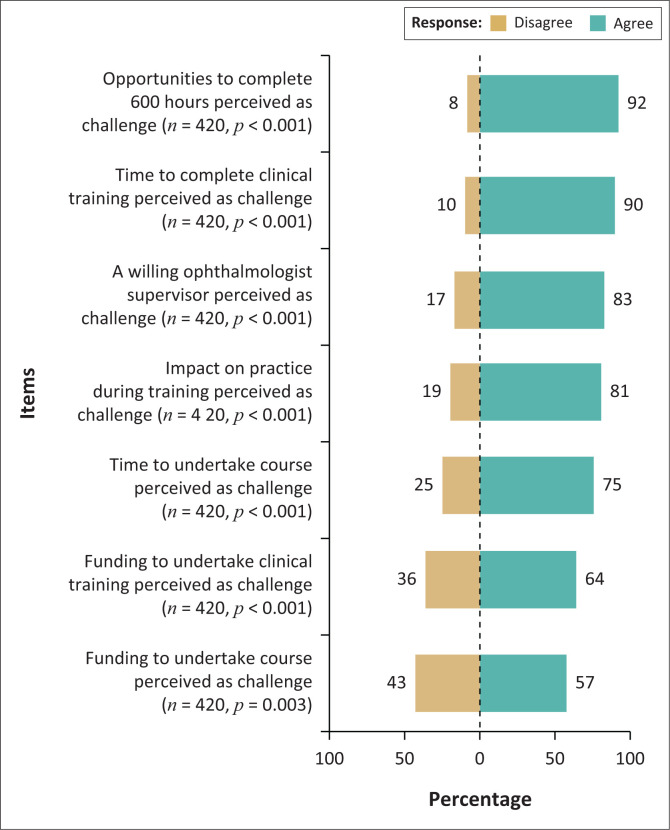
Respondents’ agreement with perceived challenges to ocular therapeutics certification.

Agreement with all perceived challenges as listed in Figure 5 was expressed by a significant majority of the respondents. Opportunities and time to complete the clinical training were perceived as the greatest challenges (Figure 5). This was also observed in the respondents’ perceptions of the required clinical training hours for PGCOT with the majority of the respondents (*n* = 259, 61.8%) reporting the minimum clinical hours required as being ‘too much’ compared with 36.5% (*n* = 153) who perceived it as being appropriate.

### Potential benefits of the optometrists using ocular therapeutics in practice

**FIGURE 6:**
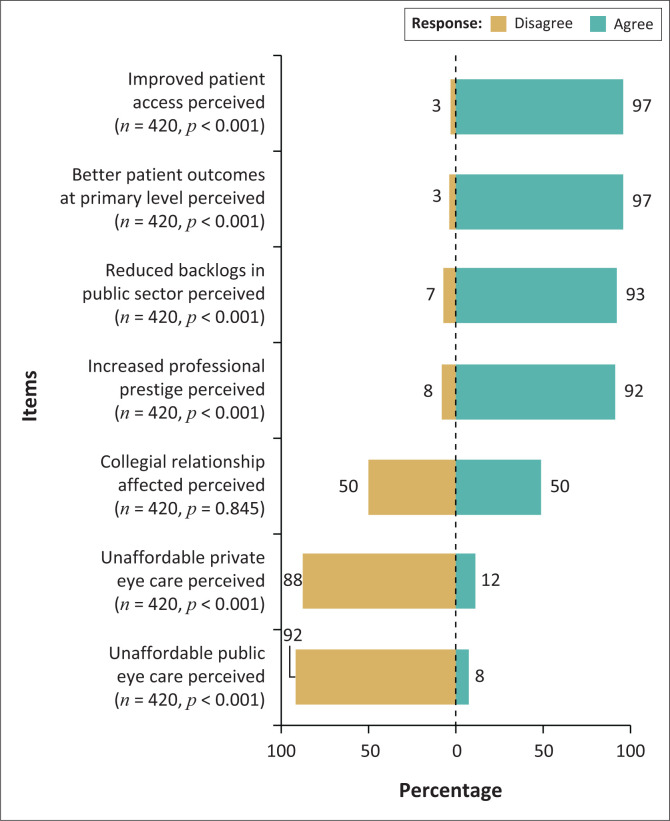
Respondents’ agreement with suggested potential benefits of the ocular therapeutics certified optometrist.

The potential benefits of an ocular therapeutics qualified optometrist, as suggested by the researchers, and the levels in agreement (% of respondents) for each statement are shown in Figure 6.

The greatest potential benefit of ocular therapeutics perceived by a significant majority of respondents was improved patient access and better patient outcomes. The majority of respondents disagreed that certification of optometrists with ocular therapeutics would make eye care unaffordable both in the private and public sector.

## Discussion

The scope of optometry worldwide has evolved tremendously in accordance with the World Health Organization (WHO) definition with the expectation that allowing optometrists to have therapeutic privileges, primary and secondary eye care could be delivered even more promptly, effectively and successfully with a possible outcome of minimising the burden of ocular diseases and preventable blindness particularly on the African continent. The inclusion of ocular therapeutics is a recent change in the scope of optometry in South Africa; however, several years later not many practitioners have qualified for this certification and this study set out to explore possible reasons for this, in addition to the perception of South African optometrists of this expanded scope, using an online survey.

A response rate of 10.4% was obtained for the online survey, with two-thirds of the respondents being female in line with the gender distribution of optometrists currently registered with the HPCSA, which reflects 61.6% being female (Daffue Y 2021, personal communication to Hansraj R, November 03, 2021). Based on the preponderance of females in the South African optometry fraternity, as well as the suggestion that females are more likely to engage on social media platforms for educational purposes,^15^ this finding cannot be taken to indicate a greater interest in ocular therapeutics among female optometrists in SA. With respect to age, the median age of the respondents was 36 years with at least half of all the respondents having graduated around 15 years ago or less. This may suggest greater interest in this topic by the younger optometrist considering the fact that ocular therapeutics have been included in the undergraduate curriculum in the more recent years. In addition, strong associations were observed between age and current usage of diagnostics procedures, as well as keenness for certification in ocular therapeutics, which may indicate that the younger optometrists in South Africa are more amenable for scope expansion to include ocular therapeutics.

These findings may also be influenced by diagnostics procedures having been included into the undergraduate curriculum in South Africa about 22 years ago giving the younger generation a relatively greater awareness of the role of optometrists in the diagnosis and management of ocular diseases. A strong association was also observed with keenness on ocular therapeutics certification and graduating institution, with most respondents having graduated with their bachelor’s degree from the UKZN. The UKZN being the first institution in SA to offer the PGCOT may have been influential in this finding, however another factor could be the regional dominance of KwaZulu-Natal practitioners in most of the social media groups used in the distribution of the survey. These were the groups that were used as they were most accessible to the researchers; however, attempts were made for a wider distribution within South Africa with the assistance of continuing practitioner development (CPD) platform and social media groups in the different provinces. It is noteworthy that the UJ had offered their first PGCOT in 2021, which could be influential in motivating optometrists outside of KZN to consider ocular therapeutics certification. Furthermore, creating awareness of the potential benefits of ocular therapeutics during formal CPD activities, non-formal buddying and peer mentorship programmes may be useful in increasing the interest and improving confidence among optometrists to include ocular therapeutics into their practice routines.

Refraction and dispensing, together with contact lenses, remain the primary skills currently practised routinely by most optometrists when compared with other specialist areas including BV and PV, with LV being the least practised regularly. These findings could suggest a lack of demand for specialist eye care or, on the other hand, could also indicate a lack of confidence in knowledge and/or skills, as well as access to necessary equipment, to venture into these specialist areas in routine practice. They could also be related to the remuneration structure of medical aids because most respondents (86.4%) were in private practice. Similar findings were reported following a study of optometric practices and practitioners in KZN, SA, which found that the majority (87%) of respondents perceived refraction and dispensing as essential categories of a successful optometric practice with lower numbers of 64%, 74% and 78% believing other specialist fields such as PV, BV and LV, respectively, to be of importance.^16^ The challenge of appropriate remuneration for these specialist skills, which often require more chair time and specialist equipment, is an area that should be explored through relevant forums with the support of the HPCSA.^17^

Most respondents in this study practised in the private sector, which could also explain refraction and dispensing being the core function of their business. Diagnostic procedures and ocular therapeutics should complement one another. However, despite 76% of respondents being diagnostics certified, only 42% practised it routinely. While this may be expected in the public sector where there may be an ophthalmologist or ophthalmic medical officer available providing these services, for the optometrist in the private sector the use of these techniques is essential for an accurate diagnosis before an appropriate ocular therapeutic management can be instituted. The higher chair time required and non-remuneration for diagnostics procedures during an optometric examination are factors that could be hindering the use of diagnostic procedures by appropriately qualified optometrists.

The majority (97.4%) of respondents believed that ocular therapeutics should be part of the optometric scope of practice supported by the finding that the majority were either keen to, had already obtained, or were in the process of obtaining their ocular therapeutics certification. These findings are similar to that reported by an independent review commissioned by the General Optical Council in Australia on optometrist therapeutic prescribing that on average optometrists showed an increased interest in therapeutic training because of an increased sense of autonomy and a desire to apply their professional skills and clinical expertise.^14^ In addition to this, practitioners felt that having the ability to perform ocular therapeutics increased their competence as healthcare practitioners. However, discouragements to undertaking non-medical prescribing training were the added responsibility that came with the ability to practise under the expanded scope as well as a lack of financial remuneration.^14^ Remuneration also surfaced as a possible factor influencing both the practice of diagnostics and ocular therapeutics in this study as the majority (92.1%) of respondents believed that there should be some form of remuneration for the additional qualification of ocular therapeutics, with procedure-specific claims being the preferred method for this issue. Some procedures that the claims could be linked to, which majority of respondents supported for inclusion into the expanded scope, include scleral indentation, punctal plug insertion and lacrimal dilation and irrigation. Notably in a Council of Medical Schemes circular of March 2021 under discussion was that optometry claims for the expanded scope were being unjustifiably rejected or short paid.^18^ Respondents, however, did not envisage remuneration of the practitioner for the expanded scope as having a negative effect on affordability for patients. The need for the additional qualification will be averted with the incorporation of ocular therapeutics training into the undergraduate optometry curriculum; however, this initiative has encountered challenges including being able to fit the didactic and clinical components required for ocular therapeutics certification into the current 4-year programme as institutions are not keen on expanding the duration of the BOptometry programme beyond 4 years. Yet it should prove more cost-effective to have optometrists trained in ocular therapeutics during their undergraduate training, which optometry departments can use as part of their motivations to their relevant institutions for support in this regard.

Respondents in this study expressed confidence on including ocular therapeutics as part of their clinical routine. This, together with the keenness expressed, reflects an understanding of how therapeutic privileges would enable them to deliver primary and secondary eye-care services more promptly and effectively.^14,19^ This assertion is corroborated by a study in the UK, which found that optometrists trained in glaucoma management could potentially manage and treat patients with ocular hypertension in the community without a referral to an ophthalmologist.^20,21^ Moreover, specially trained and accredited optometrists working independently within the hospital environment, monitoring glaucoma and glaucoma-suspected patients were seen to be capable of making clinical management decisions that were in close agreement with those of glaucoma-specialist consultant ophthalmologists.^20^ Considering that patients with conditions such as ocular hypertension and glaucoma require lifelong management usually by an ophthalmologist, and often within an institution in the public sector, the comparatively lower numbers of ophthalmologists to optometrists worldwide serve as an impediment to the availability of safe, prompt and effective treatment.^21,22^

A similar scenario is encountered in South Africa where the number of optometrists currently registered with the HPCSA exceeds that of ophthalmologists by close to seven-fold. There are approximately 4204 registered optometrists compared with 582 ophthalmologists (Daffue Y 2023, personal communication to Hansraj R, April 19, 2023) serving a population of about 60.6 million (Statistics SA, 2022). Therefore, the ocular therapeutics equipped optometrist would benefit the delivery of eye care including allowing ophthalmologists to prioritise more complicated cases such as those requiring surgical intervention. This may offer a possible solution to bridging the existing gap in the number of ophthalmologists available as human resources for addressing the eye health issues in the African region.^8^ This envisaged outcome is reflected in the report by Ansari et al.^23^ that independent prescribing specialised optometrists effectively managed a vast majority of urgent cases at a UK hospital emergency unit and mitigated the reduced availability of eye care personnel during the coronavirus disease 2019 (COVID-19) crisis. Furthermore, as optometrists are relatively more easily available than ophthalmologists especially in rural areas, having therapeutically endorsed optometrists could potentially decrease the need for unnecessary referrals.^19^ This may reduce the heavy demand on the healthcare system to provide patients with access to the basic healthcare they may require and in turn decrease backlogs at public sector eye care facilities.^19^ Such potential benefits of an ocular therapeutics-qualified optometrist were agreed on by the majority of respondents. Respondents were divided as to whether this expanded scope of optometry could affect the collegiality with ophthalmology. To the contrary, Fang and Narunsky^19^ asserted that co-management would strengthen inter-professional relationships between ophthalmology and optometry.

There appeared to be a greater preference for the usage of topical rather than oral medications. This may be related hesitancy by optometrists to prescribe medication that could have broader, including systemic, sideeffects, which explain the findings in this study of the respondents seemingly relatively less confident with the inclusion of topical anti-glaucoma agents, analgesics and anti-inflammatories as well as oral antihistamines and anti-glaucoma agents. It could also be related to optometrists possibly encountering more anterior segment pathology, particularly in the private sector. In support of these assertions, of note are the results of a survey in Australia, which revealed that the vast majority of therapeutically endorsed optometrists were confident to independently manage patients with allergic conjunctivitis, dry eye, episcleritis, infective conjunctivitis, blepharitis, meibomianitis and superficial inflammation.

Optometrists, however, showed less confidence and greater reliance on ophthalmologists for patients with glaucoma, microbial keratitis, uveitis and to a lesser extent viral keratitis as these conditions were more serious with a greater potential for irreversible vision loss.^24^ Similar prescribing rights are observed in the USA where all states allow optometrists prescribing rights for topical drugs, fewer allow for oral or intravenous medications.^19^ Most respondents in this study supported the inclusion of foreign body removal, punctal plug insertion, scleral indentation and lacrimal dilation and irrigation in the expanded optometric scope of practice. These procedures may be relatively uncomplicated and with proper training optometrists could easily be skilled in them as observed in the USA, where some states permit, optometrists to perform minor ocular surgeries including selective laser trabeculoplasty where they have achieved a fair amount of success when compared with ophthalmologists.^2,21^ The potential benefits of optometrist with certification and confidence in managing anterior segment disorders, together with being able to do minor surgical procedures such as foreign body removals and lacrimal drainage, particularly to individuals who have little to no access to ophthalmological care, are immense.

Diagnostics certification is important to execute ocular therapeutic privileges, particularly for the management of posterior segment pathology. Thus, it was encouraging to notice that most of the respondents were diagnostics certified, with just over two-thirds having obtained the necessary skills during their undergraduate training. These were mainly the younger generation of optometrists as diagnostics training was only incorporated into undergraduate programmes in SA from the year 2000. Training institutions should embark on surveys to gauge the interest in diagnostics certification by optometrists who had entered the programme prior to the year 2000, and upskilling short courses should be provided to bridge this gap of practising optometrists without diagnostic certification. This initiative in itself may provide awareness and motivation towards obtaining certification in ocular therapeutics.

A very different picture was observed for ocular therapeutics certification. While 19% of respondents had attempted and completed the theoretical component of the course, only 4% had completed the relevant clinical training. Furthermore only 10% were currently registered with the HPCSA with ocular therapeutic privileges despite having completed the theoretical modules in 2015. A similar situation was noticed in Australia where ocular therapeutics was authorised in 1996, yet because of administrative issues, optometrists had their registration endorsed only in 2000 for this expanded scope.^25^ More recently, in 2014, an online Certificate in Ocular Therapeutics (COT) course has been offered by the Australian College of Optometry from which 84 optometrists from Australia and New Zealand have graduated.

While the current undergraduate training in optometry was reported by many as providing a good baseline knowledge to attempt the PGCOT, a refresher course focused on ocular pathology was thought of as being useful by a significant majority of the respondents, prior to attempting the ocular therapeutics qualification. Unlike in other studies as reported by Carey et al.^14^ further training in pharmacology, in particular, was not perceived as being required in this study. Instead, the major challenge to obtaining ocular therapeutics certification appears to be with the required number of 600 clinic hours, which was perceived by many respondents (61.8%) as being ‘too much’. In addition, this challenge of the number of required clinical hours appears to be further exacerbated by the lack of opportunities and time to complete the required clinical training similar to the challenges reported by Carey et al.^14^ In contrast, the COT course in Australia requires optometrists to complete only 50 h of clinical placement with ophthalmologists, and 84 of 180 optometrists who enrolled have graduated already.^26^ In the UK, ‘Independent Prescriber’ registration (IP) with the General Council of Optometry GOC requires 72 h of clinical work under the supervision of an ophthalmologist in addition to the theoretical course and assessment by the College of Optometrists; however, annual renewal is required accompanied by a detailed log of prescribing activity.^14^ These discrepancies highlight the need for the current minimum of 600 clinical hours for the PGCOT to be reviewed.

Clinical training towards ocular therapeutics certification, particularly in terms of hours and placement sites, appears to be a challenge in many countries in which optometrists now have non-medical prescribing rights. Similar to the findings of this study, a survey of 60 optometrists in the UK, reported that satisfaction ratings for various components of independent prescribing training in general were high, with 75% believing training was relevant and helpful to practise.^27^ However, 25% indicated they did not have adequate exposure to relevant clinical conditions and number of patients during training, or they had minimal opportunity for discussion of prescribing decisions with ophthalmologists.

The main barriers to training were identified as difficulty in finding a hospital for clinical placement and the length of time it took for placement completion (38% took 6 months to 1 year). Furthermore, this challenge of clinical placement was highlighted by Carey et al.^14^ following a review of studies on optometrist ocular therapeutics prescribing, who reported practice-based learning under the supervision of an ophthalmologist to be problematic in terms of availability and accessibility, especially with the clinical placements. It was thus recommended that there be a review of arrangements and provision for practice placements so that more optometrists could qualify with ocular therapeutics. This appears to be a major consideration to enable ocular therapeutics certification of SA optometrists as well.

This study provided useful and novel insight into the interest and readiness of optometrists in South Africa for ocular therapeutics, however as this is the first study focussed on this area there are acknowledged limitations. While respondents to the online survey included graduates from all institutions in SA currently training optometrists, a bias may have been created as the survey was distributed largely via social media groups that included mostly KZN optometrists. Furthermore, almost 45% of the respondents were UKZN graduates who may have had more exposure to the expanded scope because of that institution being the first one in SA to offer the certification course.

The results may have also been biased by a better response rate from optometrists that are eager to obtain their ocular therapeutics certification. Even though an acceptable response rate was obtained, a wider perspective and greater response rate may be obtained if a similar survey could be distributed by the South African Optometric Association (SAOA) and/or HPCSA, which is the overarching body for education, training and registration of health professionals in SA including optometrists. The survey content was primarily close ended that may have limited a more detailed exploration of certain aspects. Thus, future surveys should include open-ended questions, as well as possibly focus group discussions.

In view of the findings of this study, current requirements to support ocular therapeutics certification of South African optometrists should be reviewed to ensure an enabling environment for the completion of the clinical training. This review should take cognisance of similar requirements in other countries where optometrists have been able to obtain non-medical prescribing rights and practise them with a fair amount of success. Academic institutions that provide post graduate training for this certification should consider refresher courses in both ocular pathology and diagnostic techniques. It is also recommended that the Professional Board for Optometry and Dispensing Opticians of the HPCSA reviews the role and scope of practice of optometrists based on a needs assessment, which can be used to better inform the process of expanding the scope of optometry.

## Conclusion

Most optometrists in South Africa display a keen interest in ocular therapeutics certification. There is a preponderance of diagnostically qualified optometrists, with a majority reporting confidence in including ocular therapeutic techniques into their daily practice. However, very few optometrists in South Africa are currently certified for this expanded scope. Limited opportunities to complete the required minimum clinical hours appears to be the main challenge to certification in South Africa. The ocular therapeutics certified optometrist is perceived as having the potential to improve patient access to beneficial eye care and ensure optimal patient health outcomes at a primary health care level.
